# Beyond the frontiers of neuronal types

**DOI:** 10.3389/fncir.2013.00013

**Published:** 2013-02-07

**Authors:** Demian Battaglia, Anastassios Karagiannis, Thierry Gallopin, Harold W. Gutch, Bruno Cauli

**Affiliations:** ^1^Department of Nonlinear Dynamics, Max Planck Institute for Dynamics and Self-Organization (MPIDS)Göttingen, Germany; ^2^Bernstein Center for Computational NeuroscienceGöttingen, Germany; ^3^CNRS UMR7102, Laboratoire de Neurobiologie des processus adaptatifs, Université Pierre et Marie CurieParis, France; ^4^Neuroscience, Physiology and Pharmacology, Division of Biosciences, University College LondonLondon, UK; ^5^CNRS UMR 7637, Laboratoire de Neurobiologie et Diversité Cellulaire, ESPCI ParisTechParis, France; ^6^Department of Mathematics, Technische Universität MünchenGarching, Germany

**Keywords:** neuronal diversity, interneuron diversity, barrel cortex, petilla terminology, atypical cells, fuzzy sets, unsupervised clustering

## Abstract

Cortical neurons and, particularly, inhibitory interneurons display a large diversity of morphological, synaptic, electrophysiological, and molecular properties, as well as diverse embryonic origins. Various authors have proposed alternative classification schemes that rely on the concomitant observation of several multimodal features. However, a broad variability is generally observed even among cells that are grouped into a same class. Furthermore, the attribution of specific neurons to a single defined class is often difficult, because individual properties vary in a highly graded fashion, suggestive of continua of features between types. Going beyond the description of representative traits of distinct classes, we focus here on the analysis of atypical cells. We introduce a novel paradigm for neuronal type classification, assuming explicitly the existence of a structured continuum of diversity. Our approach, grounded on the theory of fuzzy sets, identifies a small optimal number of model archetypes. At the same time, it quantifies the degree of similarity between these archetypes and each considered neuron. This allows highlighting archetypal cells, which bear a clear similarity to a single model archetype, and edge cells, which manifest a convergence of traits from multiple archetypes.

## Introduction

Cortical physiology and function depend on a delicate interplay between excitatory glutamatergic neurons and diverse inhibitory GABAergic interneurons in a temporally, spatially and cell-type-specific manner (Gupta et al., 2000; Somogyi and Klausberger, 2005; Sohal et al., 2009; Cauli and Hamel, 2010; Kätzel et al., 2011; Gentet, 2012). A pertinent understanding of information processing requires thus a thorough description of this neocortical neuronal diversity.

Historically, inhibitory interneurons have been subdivided according to a large repertoire of morphological, electrophysiological, and molecular properties (Ascoli et al., 2008). More recently, classification schemes based on developmental criteria largely confirmed and further supported (Butt et al., 2005; Miyoshi et al., 2007; Batista-Brito and Fishell, 2009; Vucurovic et al., 2010) previous classifications (McCormick et al., 1985; Kawaguchi and Kubota, 1993, 1996; Cauli et al., 1997). This emphasizes the concept that neuronal types can and must be defined by the convergence of common features and not solely by a limited set of prescribed properties (Tyner, 1975).

Unsupervised clustering methods have so far established themselves as the state-of-the-art approach to identify neuronal types based on the simultaneous consideration of many features (Tamás et al., 1997; Cauli et al., 2000; Toledo-Rodriguez et al., 2004; Gallopin et al., 2006; Halabisky et al., 2006; Dumitriu et al., 2007; Helmstaedter et al., 2009; Karagiannis et al., 2009; McGarry et al., 2010; Suzuki and Bekkers, 2010; Perrenoud et al., 2012a,b). Although useful, these classification schemes are hampered by at least two shortcomings. First, a distinctive trait can be shared by multiple cell types. For instance, parvalbumin (PV) expression is common to both some basket cells and chandelier cells (Kawaguchi and Kubota, 1993; Toledo-Rodriguez et al., 2004; Woodruff et al., 2010). Second, a feature discriminative for certain neuronal types can be irrelevant and highly variable for other types. Examples are spike width and input resistance, respectively, very thin and small for Fast Spiking (FS)-PV cells, but highly heterogeneous for both Somatostatin- (SOM) and Vasoactive Intestinal Polypeptide (VIP)-expressing cells (Kawaguchi and Kubota, 1996; Cauli et al., 2000; Ma et al., 2006).

Because of these shortcomings, the boundaries between different types are blurred. Yet, despite this, all clustering methods used so far for neuronal classification inherently exclude the existence of a graded separation between neuronal classes (Tyner, 1975; Parra et al., 1998). As an alternative, we propose here a novel approach, based on the theory of Fuzzy Sets (Zadeh, 1965, 2008), in which the absence of a strict dividing line between neuronal types is actually assumed as an explicit categorizing hypothesis.

By applying this approach to a well-characterized multi-parametric dataset (Karagiannis et al., 2009), we confirm the identification of four main types of inhibitory neurons (canonic *archetypes*). Beyond this, our method provides a way to quantify how individual cells are similar to these archetypes, opening the way to a systematic study of deviations from typical tendencies. In particular, we unmask atypical cells displaying properties simultaneously reminiscent of multiple archetypes. These *edge cells* might contribute to robustness and flexibility of performance in neural circuits by enriching, with their polyvalent phenotypes, the reservoir of available functional resources.

## Materials and methods

### The dataset

We performed here an alternative analysis of a dataset presented in Karagiannis et al. (2009). This previous study focused on typical properties of interneuron classes, while we emphasize here deviations from these typical aspects. The sample consisted of 200 neurons from slices from juvenile male Wistar rat somatosensory cortex (postnatal days 19 ± 2, SD, Charles River, L'Arbresle, France). Overall 43 features were measured for each neuron, including digitized value of laminar location of the soma (with non-integer values indicating cells at the border between different layers, e.g., 1.5 meaning between layers I and II), 32 electrophysiological features (5 sub-threshold, 4 just-above threshold, 5 firing, and 18 action potential properties) and expression of 10 well-established molecular markers of neuronal diversity (Ascoli et al., 2008). Morphology, reconstructed only for a limited subset of cells, was not considered in the current analysis. A complete list of the used features is reported in Tables 1–4. The reader is invited to refer to (Karagiannis et al., 2009) for all details on feature definitions and on the used experimental procedures.

**Table 1:** **Localization and subthreshold features of different archetypes**.

	**Glutamatergic**	**FS-PV**	**Adapting SOM**	**Adapting VIP**	**Adapting NPY**	**UFO**
	**(*n* = 49)**	**(*n* = 33)**	**(*n* = 21)**	**(*n* = 31)**	**(*n* = 33)**	**(*n* = 33)**
(1) Digitized laminar location (*Layer*)	**3.4 ± 0.8**	**3.1 ± 0.8**	2.8 ± 0.7	2.7 ± 0.7	**2.3 ± 0.6**	**2.2 ± 0.7**
		**UFO, Adapt. NPY** < Adapt. VIP, Adapt. SOM << **FS-PV, Glutamatergic**				
(2) Resting potential (*V*_*m*_, mV)	**−74.3 ± 4.7**	**−72.7 ± 4.0**	**−65.0 ± 3.9**	−71.0 ± 5.1	−72.2 ± 4.5	−70.8 ± 5.5
		**Glutamatergic,** FS-PV, Adapt. NPY, “UFO,” Adapt. VIP <<< **Adapt. SOM**				
		**Glutamatergic** << UFO; **Glutamatergic** < Adapt. VIP			
(3) Input resistance (*R*_*m*_, MΩ)	379 ± 138	**204 ± 77**	272 ± 76	**514 ± 166**	314 ± 116	**552 ± 266**
		**FS-PV** << Adapt. SOM << Glutamatergic << **Adapt. VIP, UFO**			
		**FS-PV** <<< Adapt. NPY <<< **Adapt. VIP, “UFO”**				
(4) Time constant (τ_*m*_, ms)	**36.1 ± 10.2**	**15.6 ± 6.0**	24.2 ± 8.7	24.6 ± 10.0	22.8 ± 8.3	29.0 ± 12.0
		**FS-PV** <<< Adapt. NPY, Adapt. VIP < UFO << **Glutamatergic**				
		**FS-PV** << Adapt. SOM < UFO				
(5) Membrane capacitance (*C*_*m*_, pF)	**104.0 ± 32.3**	79.1 ± 21.7	91.3 ± 23.4	**48.5 ± 14.3**	76.8 ± 22.7	**56.0 ± 20.3**
		**Adapt. VIP, UFO** <<< Adapt. NPY, FS-PV << **Glutamatergic**				
		**Adapt. VIP, “UFO”** <<< Adapt. SOM				
(6) Sag index (*Sag*, %)	**19.7 ± 9.2**	9.4 ± 5.3	**27.6 ± 12.0**	8.2 ± 3.7	9.0 ± 4.8	**6.4 ± 3.2**
		**UFO** < Adapt. VIP, FS-PV <<< **Glutamatergic < Adapt. SOM**				
		**UFO** << Adapt. NPY <<< **Glutamatergic < Adapt. SOM**				

*Values are weighted means ± SD; n, number of cells; < significantly smaller with P ≤ 0.05; << significantly smaller with P ≤ 0.01; <<< significantly smaller with P ≤ 0.001*.

**Table 2:** **Above threshold features of different archetypes**.

	**Glutamatergic**	**FS-PV**	**Adapting SOM**	**Adapting VIP**	**Adapting NPY**	**UFO**
	**(*n* = 49)**	**(*n* = 33)**	**(*n* = 21)**	**(*n* = 31)**	**(*n* = 33)**	**(*n* = 33)**
(7) Rheobase (*I*_rheo_, pA)	35.8 ± 26.2	**103.1 ± 46.4**	**−4.3 ± 30.2**	16.3 ± 12.7	53.0 ± 28.5	32.7 ± 22.0
		**Adapt. SOM** <<< Adapt. VIP << “UFO,” Glutamatergic << Adapt. NPY <<< **FS-PV**				
(8) First spike latency (τ_1st_, ms)	127.1 ± 46.5	370.7 ± 245.4	110.8± 88.3	125.2 ± 113.7	218.5 ± 209.9	**106.6 ± 112.2**
		**“UFO”** < Adapt. SOM < Adapt. VIP, Glutamatergic, Adapt. NPY				
		Glutamatergic, Adapt. SOM << FS-PV				
(9) Just-above threshold adaptation (Ad_thr_, Hz/s)	**−46.8 ± 74.9**	**3.3 ± 20.4**	−15.6 ± 25.9	−6.9 ± 18.4	**−0.4 ± 4.1**	**−32.6 ± 38.6**
		**Glutamatergic, “UFO”** < Adapt. VIP << **Adapt. NPY, FS-PV**				
		Adapt. SOM << **Adapt. NPY, FS-PV**				
(10) Minimal steady state frequency (*F*_min_, Hz)	30.9 ± 53.8	14.9 ± 13.3	13.7 ± 12.3	13.1 ± 10.1	**6.1 ± 3.0**	**31.7 ± 27.1**
		**Adapt. NPY** <<< Adapt. VIP, Adapt. SOM, FS-PV, **“UFO”; Adapt. NPY** << Glutamatergic				
		Adapt. VIP, Adapt. SOM < **“UFO”**				
(11) Amplitude accommodation (*A*_hump_, mV)	**24.3 ± 8.8**	**1.1 ± 1.2**	3.8 ± 4.3	5.2 ± 4.8	10.6 ± 7.4	7.1 ± 5.6
		**FS-PV** <<< Adapt. VIP, “UFO” < Adapt. NPY <<< **Glutamatergic**				
		**FS-PV** << Adapt. SOM < “UFO”; Adapt. SOM <<< Adapt. NPY				
(12) Amplitude of early adaptation (*A*_sat_, Hz)	**153.1 ± 59.4**	**52.7 ± 24.8**	93.9 ± 24.3	110.7 ± 41.3	127.4 ± 37.0	109.8 ± 37.1
		**FS-PV** <<< Adapt. SOM, “UFO,” Adapt. VIP << **Glutamatergic**				
		Adapt. SOM << Adapt. NPY < **Glutamatergic**				
(13) Time constant of early adaptation (τ_sat_, ms)	25.7 ± 11.6	**18.4 ± 15.9**	**37.8 ± 7.8**	23.9 ± 8.8	25.8 ± 6.1	27.1 ± 8.8
		**FS-PV** < Adapt. VIP, Adapt. NPY, Glutamatergic, “UFO” <<< **Adapt. SOM**				
(14) Late adaptation (Ad_sat_, Hz/s)	**−9.6 ± 7.3**	−27.6 ± 13.1	−20.6 ± 9.2	**−35.6 ± 11.3**	−21.3 ± 10.0	26.1 ± 14.9
		**Adapt. VIP** < FS-PV, “UFO,” Adapt. NPY, Adapt. SOM <<< **Glutamatergic**				
		**Adapt. VIP** << “UFO”; **Adapt. VIP** <<< Adapt. NPY, Adapt. SOM				
(15) Maximal steady state frequency (*F*_max_, Hz)	**29.9 ± 8.3**	**140.4 ± 30.4**	69.1 ± 19.9	87.0 ± 29.2	61.1 ± 13.9	64.3 ± 22.0
		**Glutamatergic** <<< Adapt. NPY, “UFO” << Adapt. VIP <<< **FS-PV**				
		**Glutamatergic** <<< Adapt. SOM <<< **FS-PV**				

*Values are weighted means ± SD; n, number of cells; < significantly smaller with P ≤ 0.05; << significantly smaller with P ≤ 0.01; <<< significantly smaller with P ≤ 0.001*.

**Table 3:** **Action potentials features of different archetypes**.

	**Glutamatergic**	**FS-PV**	**Adapting SOM**	**Adapting VIP**	**Adapting NPY**	**“UFO”**
	**(*n* = 49)**	**(*n* = 33)**	**(*n* = 21)**	**(*n* = 31)**	**(*n* = 33)**	**(*n* = 33)**
(16) First spike amplitude (*A*_1_, mV)	93.0 ± 7.2	**81.9 ± 7.5**	94.9 ± 7.6	**98.0 ± 6.5**	93.4 ± 5.7	91.5 ± 13.0
		**FS-PV** <<< Glutamatergic, Adapt. NPY, Adapt. SOM << **Adapt. VIP**				
		**FS-PV** << “UFO” < **Adapt. VIP**				
(17) Second spike amplitude (*A*_2_, mV)	85.2 ± 11.7	**79.1 ± 8.0**	90.7 ± 7.7	92.7 ± 7.5	90.0 ± 5.7	**79.9 ± 9.3**
		**FS-PV, “UFO”** <<< Adapt. NPY, Adapt. SOM, Adapt. VIP				
		**FS-PV, “UFO”** < Glutamatergic << Adapt. VIP				
(18) First spike duration (*D*_1_, ms)	**1.4 ± 0.2**	**0.6 ± 0.2**	0.9 ± 0.2	0.8 ± 0.2	1.0 ± 0.2	1.0 ± 0.3
		**FS-PV** <<< Adapt. VIP <<< Adapt. NPY, “UFO” <<< **Glutamatergic**				
		Adapt. VIP < Adapt. SOM <<< **Glutamatergic**				
(19) Second spike duration (*D*_2_, ms)	**1.6 ± 0.3**	**0.6 ± 0.2**	1.0 ± 0.2	0.8 ± 0.2	1.1 ± 0.2	1.1 ± 0.3
		**FS-PV** <<< Adapt. VIP <<< Adapt. NPY, “UFO” <<< **Glutamatergic**				
		Adapt. VIP << Adapt. SOM <<< **Glutamatergic**				
(20) Amplitude Reduction (Δ(Amp), %)	−8.4 ± 9.7	**−1.2 ± 6.5**	−4.8 ± 3.7	−5.3 ± 4.9	−3.4 ± 2.7	−11.8 ± 9.7
		**FS-PV** <<< Adapt VIP < **“UFO”; FS-PV** <<< Glutamatergic				
		**FS-PV** << Adapt. NPY <<< **“UFO”**; Adapt. NPY < Glutamatergic				
		**FS-PV** << Adapt. SOM < **“UFO”**				
(21) Duration Increase (Δ(Dur), %)	10.7 ± 12.0	**1.3 ± 5.3**	7.4 ± 4.6	2.8 ± 4.3	9.0 ± 7.4	**12.9 ± 10.1**
		**FS-PV** << Adapt. VIP <<< Adapt. SOM, Adapt. NPY, “UFO”				
		**FS-PV** <<< Glutamatergic; Adapt. VIP << Glutamatergic				
(22) First spike, first component AHP (AHP_f_, mV)	**−6.7 ± 3.7**	**−23.2 ± 3.1**	−11.4 ± 3.8	−14.1 ± 3.8	−13.7 ± 4.4	−13.1 ± 4.6
		**FS-PV** <<< Adapt. VIP, Adapt. NPY, “UFO,” Adapt. SOM <<< **Glutamatergic**				
(23) First spike, second component AHP (AHP_s_, mV)	−8.0 ± 6.9	−2.1 ± 6.3	−6.5 ± 4.2	−8.6 ± 3.2	**−15.3 ± 3.8**	**−1.9 ± 4.2**
		**Adapt. NPY** <<< Adapt. VIP < Adapt. SOM << FS-PV <<< **“UFO”**				
		**Adapt. NPY** <<< Glutamatergic < FS-PV				
(24) Second spike, first component AHP (AHP_f,2_, mV)	**−8.8 ± 4.1**	**−23.3 ± 3.4**	−10.3 ± 6.6	−15.5 ± 4.1	−15.3 ± 4.0	−15.0 ± 5.0
		**FS-PV** <<< Adapt. NPY << Adapt. SOM < **Glutamatergic**				
		**FS-PV** <<< Adapt. VIP, “UFO” < Adapt. SOM				
(25) Second spike, second component AHP (AHP_s,2_, mV)	**−14.7 ± 8.8**	**−2.1 ± 5.7**	−7.5 ± 3.9	−10.7 ± 2.6	**−13.2 ± 8.3**	**−2.8 ± 5.3**
		**Glutamatergic** <<< Adapt. VIP <<< **“UFO,” FS-PV**				
		**Adapt.** NPY << Adapt. VIP << Adapt. SOM << **“UFO,” FS-PV**				
(26) First spike, first AHP component latency (τ_AHPf_, ms)	**14.2 ± 20.7**	**2.9 ± 1.3**	3.2 ± 1.1	**2.7 ± 0.7**	4.7 ± 1.7	4.2 ± 2.2
		**Adapt. VIP, FS-PV** <<< “UFO” <<< **Glutamatergic**				
		**Adapt. VIP, FS-PV** <<< Adapt. NPY << **Glutamatergic**				
		**FS-PV** < Adapt. SOM <<< Adapt. NPY				
(27) First spike, second AHP component latency (τ_AHPs_, ms)	46.0 ± 42.3	**1.1 ± 3.1**	27.5 ± 16.8	34.0 ± 17.2	20.1 ± 6.8	**5.1 ± 12.1**
		**FS-PV, “UFO”** <<< Adapt. NPY, Adapt. SOM, Adapt. VIP, Glutamatergic				
		Adapt. NPY <<< Adapt. VIP				
(28) Second spike, first AHP component latency (τ_AHPf,2_, ms)	**18.9 ± 23.5**	**2.9 ± 1.3**	3.4 ± 1.0	**2.8 ± 0.7**	7.0 ± 4.4	5.7 ± 4.2
		**Adapt. VIP, FS-PV** <<< “UFO” <<< **Glutamatergic**				
		**Adapt. VIP, FS-PV,** Adapt. SOM <<< Adapt. NPY << **Glutamatergic**				
		**FS-PV** << Adapt. SOM << “UFO”				
(29) Second spike, second AHP component latency (τ_AHPs,2_, ms)	**59.3 ± 38.6**	**1.1 ± 3.1**	30.1 ± 16.9	31.4 ± 14.0	16.4 ± 9.6	10.3 ± 18.6
		**FS-PV** <<< Adapt. NPY <<< Adapt. VIP << **Glutamatergic**				
		**FS-PV** <<< Adapt. SOM <<< **Glutamatergic**; Adapt. NPY < Adapt. SOM				
		“UFO” <<< Adapt. VIP, **Glutamatergic**; “UFO” << Adapt. SOM; “UFO” < Adapt. NPY				
(30) First spike ADP (ADP, mV)	1.3 ± 1.8	0.5 ± 1.5	**4.7 ± 2.5**	**7.9 ± 3.4**	0.6 ± 0.7	0.3 ± 0.8
		“UFO,” FS-PV, Adapt. NPY, Glutamatergic <<< **Adapt. SOM, Adapt. VIP**				
		“UFO,” FS-PV << Adapt. NPY; “UFO” << Glutamatergic				
(31) Second spike ADP (ADP_2_, mV)	0.3 ± 0.6	0.5 ± 1.6	3.7 ± 2.1	**7.0 ± 2.8**	0.2 ± 0.4	0.7 ± 2.0
		Adapt. NPY, Glutamatergic, FS-PV, “UFO” <<< Adapt. SOM <<< **Adapt. VIP**				
(32) First spike ADP latency (τ_ADP_, ms)	5.6 ± 6.1	**0.7 ± 1.9**	**10.4 ± 5.7**	**9.8 ± 2.7**	5.1 ± 3.5	**1.5 ± 3.9**
		**FS-PV, “UFO”** << Adapt. NPY <<< **Adapt. VIP, Adapt. SOM;**				
		**FS-PV, “UFO”** << Glutamatergic				
		Glutamatergic << **Adapt. VIP**; Glutamatergic < **Adapt. SOM**				
(33) Second spike ADP latency (τ_ADP2_, ms)	3.6 ± 4.2	**0.6 ± 1.7**	**8.5 ± 4.2**	**9.8 ± 2.1**	3.0 ± 3.6	1.8 ± 4.1
		**FS-PV** << Glutamatergic <<< **Adapt. SOM, Adapt. VIP**				
		“UFO,” Adapt. NPY <<< **Adapt. SOM, Adapt. VIP**; FS-PV << Adapt. NPY				

*Values are weighted means ± SD; n, number of cells; < significantly smaller with P ≤ 0.05; << significantly smaller with P ≤ 0.01; <<< significantly smaller with P ≤ 0.001*.

**Table 4:** **Occurrence of molecular markers in different archetypes**.

	**Glutamatergic**	**FS-PV**	**Adapting SOM**	**Adapting VIP**	**Adapting NPY**	**“UFO”**
	**(*n* = 49)**	**(*n* = 33)**	**(*n* = 21)**	**(*n* = 31)**	**(*n* = 33)**	**(*n* = 33)**
(34) VGluT1	**99%**	26%	22%	7%	34%	21%
		Adapt. VIP, “UFO,” Adapt. SOM, FS-PV, Adapt. NPY <<< **Glutamatergic**				
(35) GAD	3%	**100%**	**100%**	**100%**	**100%**	**100%**
		Glutamatergic <<< **FS-PV, Adapt. SOM, Adapt. VIP, “UFO,” Adapt. NPY**				
(36) NOS-1	0%	8%	3%	2%	23%	5%
		Glutamatergic < Adapt. NPY				
(37) CB	42%	**58%**	**77%**	8%	4%	6%
		Adapt. NPY, “UFO,” Adapt. VIP <<< **FS-PV, Adapt. SOM**				
		Adapt. NPY, “UFO” <<< Glutamatergic < **Adapt. SOM**				
		Adapt. VIP << Glutamatergic				
(38) PV	32%	**100%**	27%	12%	26%	16%
		Adapt. VIP, “UFO,” Adapt. NPY, Adapt. SOM, Glutamatergic <<< **FS-PV**				
(39) CR	2%	2%	19%	22%	11%	24%
		Glutamatergic, FS-PV < Adapt. VIP, “UFO”				
(40) NPY	2%	29%	63%	14%	**83%**	24%
		Glutamatergic, Adapt. VIP, “UFO,” FS-PV <<< **Adapt. NPY**				
		Glutamatergic << “UFO,” FS-PV, Adapt. SOM				
		Adapt. VIP << Adapt. SOM				
(41) VIP	3%	2%	5%	**92%**	4%	**55%**
		FS-PV, Glutamatergic, Adapt. NPY, Adapt. SOM <<< **“UFO” < Adapt. VIP**				
(42) SOM	2%	6%	**88%**	0%	0%	9%
		Adapt. VIP, Adapt. NPY, Glutamatergic, FS-PV, “UFO” <<< **Adapt. SOM**				
(43) CCK	7%	0%	2%	10%	2%	12%

*Values are weighted probabilities of expression; n, number of cells; > significantly larger with P ≤ 0.05; >> significantly larger with P ≤ 0.01; >>> significantly larger with P ≤ 0.001*.

### Fuzzy sets and partitions

**Figure 1:**
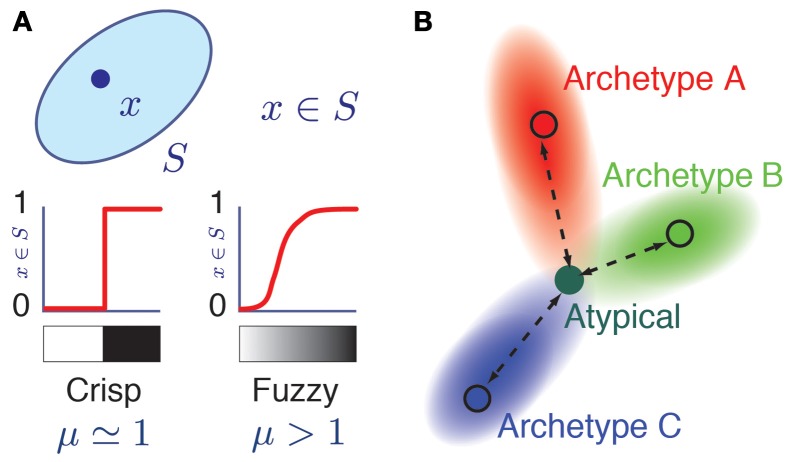
**Fuzzy membership to a class. (A)** Grayscale representation of the membership relation *x* = *S* in ordinary (left) and fuzzy (right) set theories. Here, μ is a fuzziness parameter. When μ = 1, membership relations are crisp as in ordinary set theory, while for μ > 1, membership is a soft relation. The two possible values of a *crisp* membership are represented by white (*m*_*S*_(*x*) = 0, *x* does not belong to S) and black nuances (*m*_*S*_(*x*) = 1). The graded membership in fuzzy sets (0 < *m*_*S*_(*x*) < 1) is represented by a grayscale gradient. **(B)** Color coded representation of an atypical cell having memberships with different archetypes. Its hue corresponds to a triple membership between archetypes A (Red), B (Green), and C (Blue) archetypes in the RGB color model.

In Fuzzy Set Theory (Zadeh, 1965; Jang et al., 1997; Xu et al., 2008), a data-point *i* can belong to a fuzzy class α with different degrees of membership, quantified by a membership value *m*_*i*α_. A membership value *m*_*i*α_ = 1.0 indicates that the data-point *i* displays all the defining attributes of the class α and therefore fully belongs to it. A membership value *m*_*i*α_ = 0.0 indicates that the data-point *i* does not display any of the defining attributes of the class α and therefore it does not belong to it at all. In addition to these “black and white” cases, and extending ordinary set theory, intermediate membership values 0 < *m*_*i*α_ <1 are admitted (Figure 1A), corresponding to a continuum of “gray” cases in which the data-point *i* displays only some of the defining attributes of class α. Fuzzy Set Theory thus provides, in the words of its initiator, “a precise language to describe imprecise similarities” (Zadeh, 2008).

A dataset can be partitioned into *c* fuzzy classes. In a fuzzy partition each data-point can belong to multiple classes with different degrees of membership. Formally, a fuzzy partition of a dataset with elements *i* = 1 … *N* into *c* fuzzy clusters is uniquely determined by a set of *N* membership vectors ***m***_*i*_ with *c* components each. The α-th entry *m*_*i*α_ of the vector ***m***_*i*_ gives the membership of the data-point *i* to the α-th class in the fuzzy partition. The memberships vectors are normalized in such a way that ∑^*c*^_α = 1_*m*_*i*α_ = 1.

### Unsupervised fuzzy clustering

**Figure 2:**
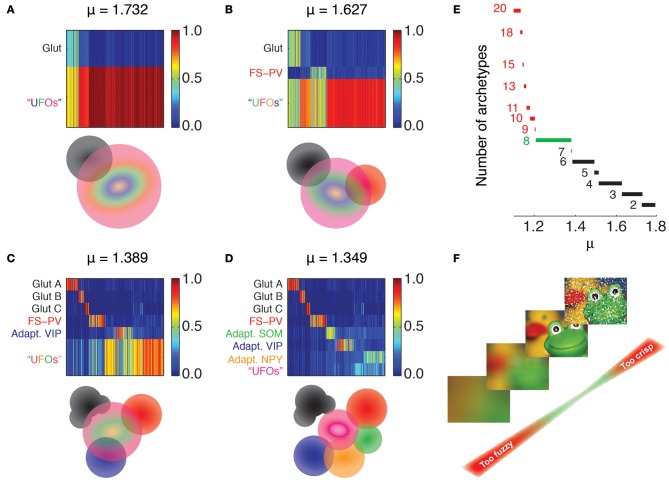
**Emergence of archetypes.** Fuzzy partitions with decreasing μ values are visualized as membership matrices (**A–D**, upper panels). Rows correspond to different fuzzy clusters and columns to individual neurons. Membership values of single neurons to each class are color coded (right bars). Schematic representation of the interrelations between archetypes (lower panels). Colored discs depict different archetypes and their overlaps denote cells with shared memberships. The Glutamatergic archetype is the first to emerge (black, **A**), followed by FS-PV interneurons (Red, **B**), and Adapting VIP interneurons (blue, **C**). Note that the glutamatergic archetype splits into three sub-groups. Adapting SOM (green) and Adapting NPY (orange) archetypes and a residual UFO archetype of highly atypical inhibitory interneurons are the last groups to singularize **(D)**. **(E)** Effective number of clusters generated by different fuzziness parameters. The number of archetypes included in the partition is indicated to the left of the corresponding range of μ. Partitions with more than eight archetypes can be only retrieved within very narrow ranges of low μ values (red). The range leading to classification with the largest number of robust archetypes is marked in green. **(F)** Metaphoric example illustrating the impact of fuzziness on the relevance of partitions and numbers of archetypes. Fuzzy partitions with too few archetypes (large μ, bottom) convey a too blurred image of the dataset. Conversely, fuzzy partitions with too many archetypes (small μ, top) are scarcely representative being strongly affected by outliers. Such issue is graphically represented as impulse noise on the image.

**Figure 3:**
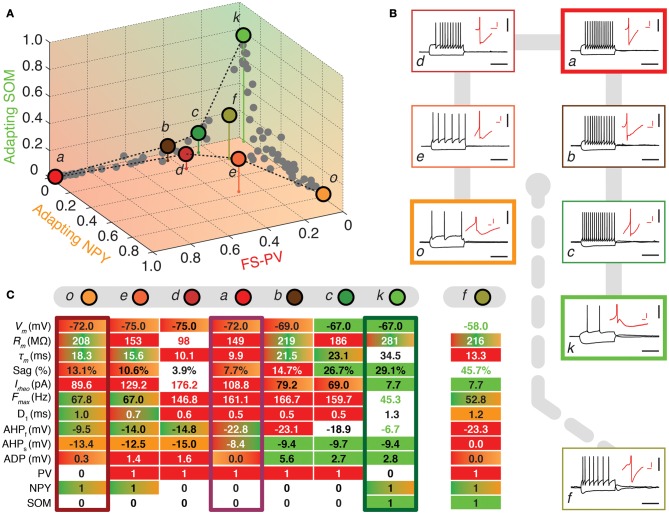
**Walking outward from the FS-PV archetype. (A)** Tetrahedral representation linking FS-PV (red), Adapting NPY (orange), and Adapting SOM archetypes (green). Individual neurons are depicted as dots in the 3D space of memberships. The X, Y, and Z axes correspond to memberships to the FS-PV, Adapting NPY, and Adapting SOM archetypes, respectively. Vertical stems denoting the projection of selected cells on the bidimensional base plane are introduced as depth clues. **(B)** Current-clamp recordings of illustrative transition neurons (cells *a* to *o* colored in panel **A**) in response to rheobase current and to a 100 pA hyperpolarizing current pulse (black traces, scale bars 50 mV, 400 ms). Insets: details of the repolarization phase of the first spike (red traces, scale bars 5 mV, 20 ms). **(C)** Table summarizing 10 electrophysiological and 3 molecular discriminative properties of transition cells between the Adapting NPY, the Adapting SOM, and the FS-PV archetypes. Orange, green, and red backgrounds indicate distinctive values for the Adapting NPY, the Adapting SOM, or the FS-PV archetype, respectively. Gradient backgrounds indicate values falling in a range typical for multiple. Bold colored entries indicate extreme values for an archetypal trend. Thick contours highlight columns corresponding to archetypal cells. Atypical cells display a heterogeneous mixture of property values which are not compatible with a single archetype or which fall in transition ranges.

**Figure 4:**
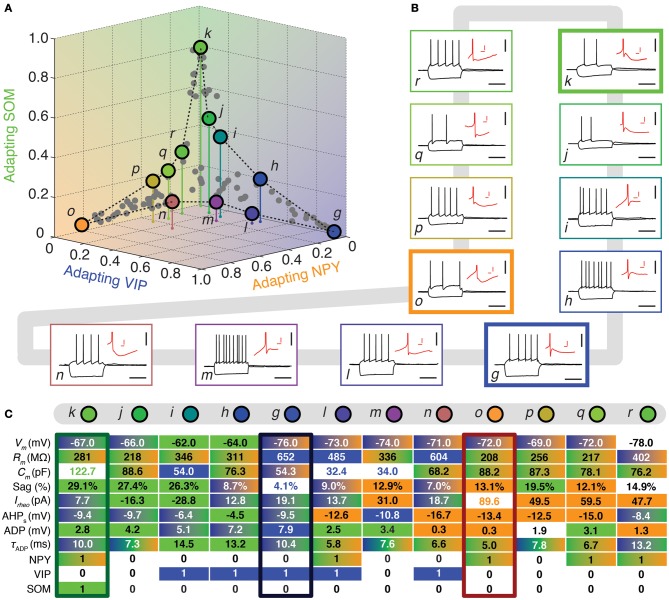
**Walking between Adapting GABAergic archetypes. (A)** Tetrahedral representation linking Adapting VIP (blue), Adapting SOM (green), and Adapting NPY (orange) archetypes. Individual neurons are depicted as dots in the 3D space of memberships. The X, Y, and Z axes correspond to memberships to the Adapting VIP, Adapting NPY, and Adapting SOM archetypes, respectively. Vertical stems denoting the projection of selected cells on the bidimensional base plane are introduced as depth clues. **(B)** Current-clamp recordings of illustrative transition neurons (cells *g* to *r* colored in panel **A**) in response to rheobase current and to a 100 pA hyperpolarizing current pulse (black traces, scale bars 50 mV, 400 ms). Insets: details of the repolarization phase of the first spike (red traces, scale bars 5 mV, 20 ms). **(C)** Table summarizing 8 electrophysiological and 3 molecular discriminative properties of transitions cells between the Adapting VIP, the Adapting SOM, and the Adapting NPY archetypes. Blue, green, and orange backgrounds indicate distinctive values for the Adapting VIP, the Adapting SOM, or the Adapting NPY archetype, respectively. Gradient backgrounds indicate values falling in a range typical for multiple. Bold colored entries indicate extreme values for an archetypal trend. Thick contours highlight columns corresponding to archetypal cells. Atypical cells display a heterogeneous mixture of property values which are not compatible with a single archetype or which fall in transition ranges.

**Figure 5:**
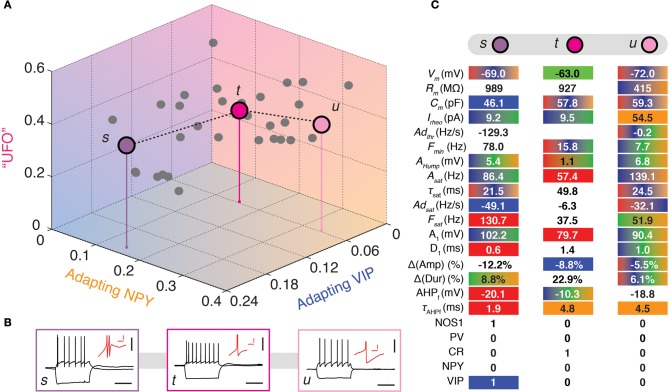
**Visiting the UFOs. (A)** Tridimensional representation joining three heterogeneous UFOs. Adapting VIP (blue), Adapting NPY (orange), and UFOs (pink) archetypes. Individual neurons are depicted as dots in a 3D space of memberships. The X, Y, and Z axes correspond to memberships to the Adapting VIP, Adapting NPY, and UFOs archetypes, respectively. Vertical stems denoting the projection of selected cells on the bidimensional base plane are introduced as depth clues. **(B)** Current-clamp recordings of illustrative transition neurons (cells *s* to *u* colored in panel **A**) in response to rheobase current and to a 100 pA hyperpolarizing current pulse (black traces, scale bars 50 mV, 400 ms). Insets: details of the repolarization phase of the first spike (red traces, scale bars 5 mV, 20 ms). **(C)** Table summarizing 17 electrophysiological and 5 molecular properties reminiscent of FS-PV (Red), Adapting VIP (blue), Adapting SOM (Green), or Adapting NPY (orange) archetypes in three different UFOs. Colored backgrounds indicate values falling in ranges typical for an archetype. Gradient backgrounds indicate values falling in a range typical for multiple archetypes.

**Figure 6:**
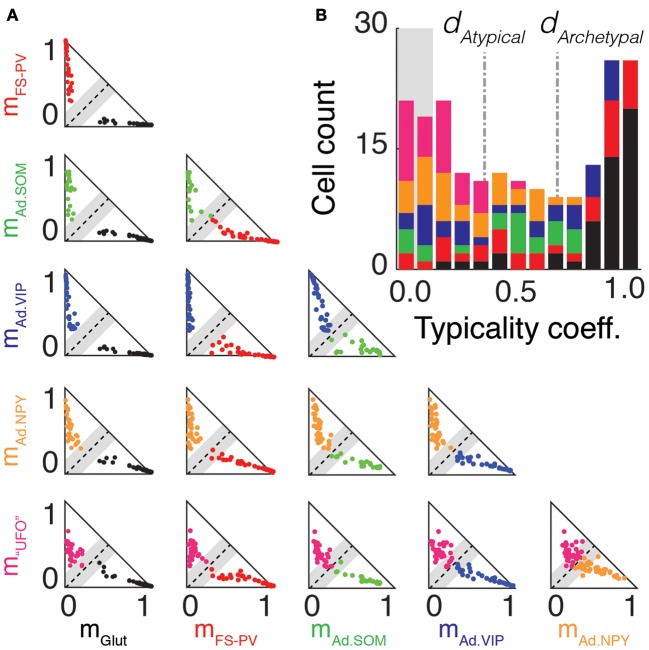
**Archetype segregations. (A)** Pairwise comparisons of archetype segregations. Two-dimensional projections of memberships of neurons belonging to the Glutamatergic (black), FS-PV (red), Adapting SOM (green), Adapting VIP (blue), Adapting NPY (orange), and UFO (magenta) archetypes. Dashed lines represent identical memberships and gray zones the mean absolute deviation of typicality. Neurons falling within the gray zone correspond to “edge cells.” Note the absence of “edge cells” between, Glutamatergic, FS-PV, and Adapting VIP neurons. **(B)** Overall distribution of typicality coefficients. The right and left peaks correspond to archetypal and atypical cells, respectively. The grayed background denotes the range of typicalities associated to edge cells. In the stacked histogram, sections with different colors indicate cells with different main type. Archetypal and atypical cells are unequally distributed across archetypes. The bimodal distribution indicates that archetypes tend to separate, but only imperfectly.

Each cell *i* (*i* = 1 … *N*) is represented as a vector of features ***f***_*i*_ with entries *f*_1_ where the index ℓ = 1 … 43 runs over the considered features (see rows of Tables 1–4). Each entry corresponds to the centered and reduced value of the corresponding feature (Cauli et al., 2000; Karagiannis et al., 2009). An unsupervised clustering algorithm was used to determine an optimized fuzzy partition of the dataset. We resorted to the fuzzy *c*-means algorithm (Dunn, 1973; Jang et al., 1997; Xu et al., 2008), a fuzzy analog of the crisp *k*-means algorithm previously used for unsupervised classification of interneurons (Karagiannis et al., 2009). This algorithm has two adjustable parameters. The first parameter is the maximally allowed number of classes, *c*. Analogously to *k*-means, the algorithm attempts to partition the dataset into *c* different fuzzy clusters. However, unlike in *k*-means, some fuzzy clusters can actually “compenetrate” and finally coalesce, leading to a fuzzy partition with a smaller effective number of clusters. We assumed here a quite large potential number of archetypes, *c* = 20. The second parameter is the fuzziness parameter μ > 1, allowing to control the level of fuzziness of the obtained partition. In the limit of μ approaching unit from higher values, the fuzzy *c*-means algorithm is equivalent to *k*-means for *k* = *c*. Larger values of this parameter lead to increasingly fuzzier partitions, with individual data-points sharing their membership between a smaller number of effective fuzzy classes. The fuzziness parameter was varied in the range 1 < μ < 2 for the analysis of Figure 2 and set to an optimal value of μ = 1.349 for the rest of the analyses in Figures 3–6 (see below for criteria guiding the choice of μ).

The fuzzy *c*-means algorithm builds a fuzzy partition of the neuronal dataset through an iterative optimization process. At a given iteration *t*, *c* cluster centroids are given by vectors **u**^(*t*)^_α_ (α = 1 … *c*) with components *u*^(*t*)^_αℓ_. Associated membership vectors **m**^(*t*)^_*i*_ are computed as:

(1) $$\frac{1}{m_{i\alpha }^{\left(t\right)}}=\sum_{\lambda =1}^{c}{\left(\frac{d_{i\alpha }^{\left(t\right)}}{d_{i\lambda }^{\left(t\right)}}\right)}^{\frac{2}{\mu -1}}$$

where *d*^(*t*)^_*i*λ_ is the Euclidean distance between the data-point ***f***_*i*_ and the centroid ***u***^(*t*)^_λ_.

These membership vectors are used in turn to compute a new set of cluster centroids ***u***^(*t* + 1)^_α_ with coordinates:

(2) $$u_{\alpha \ell }^{\left(t+1\right)}=\frac{\sum_{i=1}^{N}{\left(m_{i\alpha }^{\left(t\right)}\right)}^{\mu }f_{i\ell }}{\sum_{i\;=\;1}^{N}{\left(m_{i\alpha }^{\left(t\right)}\right)}^{\mu }}$$

This procedure is designed to minimize a specific *cost function* (Dunn, 1973; Jang et al., 1997), namely the sum of the squared distances of the data-points from the different centroids, weighted by the relative fuzzy memberships:

(3) $$J^{\left(t\right)}=\sum_{i=1}^{N}\sum_{\lambda =1}^{c}{\left(m_{i\lambda }^{\left(t\right)}\right)}^{\mu }\cdot {\left(d_{i\lambda }^{\left(t\right)}\right)}^{2}$$

In practice, as a first step, we randomly initialized a collection of *c* cluster centroids ***u***^(0)^_α_ in the feature space, by selecting *c* arbitrary data-points ***f***_*i*_. Initial membership vectors ***m***^(0)^_*i*_ were then computed using Equation (1). Equations (1) and (2) were then iterated until the positions of the *c* centroids converged to a fixed point (with a prescribed tolerance) or until a fixed maximal number of iterations was reached. The final set of centroids was then inspected to identify potential coalescences and drop redundant centroids. Whenever, the Euclidean distances between different centroids was smaller than a tolerance threshold (conventionally set to ε = 0.001), the associated fuzzy classes were merged, and the membership vectors of data-points correspondingly shrunk to a length c^*^ < *c*, by adding up memberships of the merged classes. Thus, given a dataset and a maximum number of allowed clusters *c*, the effective number c^*^ of clusters in the final fuzzy partition depended on μ. The larger μ was, the more coalescences occurred (Figure 2E).

Note that fuzzy *c*-means, as classic *k*-means, can converge to different partitions for different initial conditions. We therefore used a seeding pre-initialization strategy (see Basu et al., 2002), by selecting 7 out of 20 initial centroids to match data-points close to the centroids of the 7 *k*-means clusters analyzed in Karagiannis et al. (2009), to obtain fuzzy partitions maximally correlated with our previous crisp analyses.

### Typicality quantification

We denoted as archetypal classes of diversity (or, briefly, *archetypes*) the c^*^ fuzzy classes in an optimized fuzzy partition. Given a set of archetypes and membership vectors for a prescribed fuzziness parameter μ, the main type of a cell *i* was the archetype α with the largest membership component *m*_*i*α_ = *m*^(1st)^_*i*_. Denoting then as *m*^(2nd)^_*i*_ the second largest component of ***m***_*i*_ (associated to a secondary type), we defined the typicality coefficient of *i* as

(4) $$d(i)=m_{i}^{\left(1\text{st}\right)}-m_{i}^{\left(2\text{nd}\right)}$$

The typicality coefficient *d* is bounded in the interval 0 ≤ *d* ≤ 1.

The sample average $\bar{d}$ of typicality coefficients and their dispersion, quantified robustly by the halved mean absolute deviation Δ = *d* − *d*/2 (Sachs, 1984), were quantified over the entire sample. Based on the observed distribution of typicality coefficients (Figure 6B), cells were then considered as *archetypal* if $d>d_{\text{Archetypal}}=\bar{d}+\Delta$ and as *atypical* if $d<d_{\text{Atypical}}=\bar{d}-\Delta$.

Finally, we denoted as *edge cells* atypical neurons with a very small typicality coefficient such that *d* ≤ Δ, reflecting very similar memberships to the main and to another secondary type.

### Characterization of archetypal properties

Properties of different archetypes (cf. Tables 1–4) were computed as averages over the features of cells with a given main type, weighted by memberships toward this main type. Standard deviations were also evaluated using weighted expressions (Taylor, 1997). Defining *I*_α_ as the subset of cells having archetype α as main type, we computed, for each feature ℓ and each archetype α:

(5) $$\bar{f_{\ell }}(\alpha )=\frac{\sum_{i\in I_{\alpha }}m_{i\alpha }f_{i\ell }}{\sum_{i\in I_{\alpha }}m_{i\alpha }}$$

(6) $$\sigma_{\ell }(\alpha )=\sqrt{\frac{\left(\sum_{i\in I_{\alpha }}m_{i\alpha }\right)\cdot \sum_{i\in I_{\alpha }}m_{i\alpha }{\left(f_{i\ell }-\bar{f_{\ell }}(\alpha )\right)}^{2}}{{\left(\sum_{i\in I_{\alpha }}m_{i\alpha }\right)}^{2}-\sum_{i\in I_{\alpha }}m_{i\alpha }^{2}}}$$

These weighted averages and standard deviations reflected dominantly feature values of archetypal cells and damped the contribution of atypical cells. The *typical range* of a feature ℓ for an archetype α was defined accordingly as the interval $\bar{f_{\ell }}(\alpha )-\sigma_{\ell }(\alpha )\leq f\leq \bar{f_{\ell }}(\alpha )+\sigma_{\ell }(\alpha )$ and such convention was used to determine the coloring of table entries in the panel **(C)** of Figures 3–5.

Properties were then compared between every pair of archetypes α and β, by looking for differences between the distributions of features over the subsamples *I*_α_ and *I*_β_. Significance of pairwise comparisons was tested using the (two-tailed) Mann–Whitney U non-parametric test for continuous-valued features (i.e., lamination and electrophysiological properties) and Fisher's exact test for Boolean features (i.e., expression of molecular markers). All significant comparisons are listed in Tables 1–4.

### Relevance of different properties for classification

To analyze the impact of different properties on the quality of the extracted fuzzy partition of our dataset we compared the cost of our fuzzy partition (i.e., the value of the cost function *J*^(*t*_end_)^ as given by Equation (3) evaluated at the final iteration *t*_end_ of the clustering algorithm) with the costs of partitions extracted from partially randomized datasets.

To perform the randomization, the dataset was represented as a matrix with 43 columns, corresponding to different features, and 200 rows, corresponding to different neurons. We built randomized datasets by permuting randomly and independently the order of entries within selected columns. Such a random scrambling does not alter means and standard deviations of the randomized properties but it does disrupt their correlations with other features. The randomized datasets were then clustered with fuzzy *c*-means, using the same parameters as for the original dataset. A loss in the quality of the extracted fuzzy partition was quantified by measuring the increase of the associated cost function *J*^(*t*_end_)^. The larger the increase in residual cost after randomization of a given subset of properties, the more this property subset is considered to contribute to the quality of the reference classification.

To estimate the increase Δ*J* of the cost function due to randomization, the mean, and standard deviation of the achieved cost variation were taken over 1000 independent randomizations of each considered property subset.

This analysis is analogous to the one introduced in Karagiannis et al. (2009). In the present study, however, we quantify losses in classification quality by monitoring increases of fuzzy cost function, rather than decreases of average silhouette (Rousseeuw, 1987). This is due to the fact that standard silhouette analysis does not take into account the membership profiles of different cells.

Different features were ranked in order of decreasing Δ*J*. Fuzzy clustering was then performed taking into account only the top *K* highest ranked features, to extract reduced classifications based on a smaller number of relevant properties. The same *c* and the same seeding were always used for pre-initialization at every number of included properties *K* in order to guarantee the extraction of comparable fuzzy clusters. The fuzzy clusters in the reduced fuzzy partitions were mapped to the archetypes of the reference classification. More specifically, a fuzzy cluster α_reduced_ in a reduced classification was matched to the archetype α_ref_ in the original classification with the largest mutual overlap. For each achetype, a classification matching fraction was then defined as the percentage of cells whose original main type α_ref_ coincided with its main type α_reduced_ in the considered reduced classification.

## Results

### Emerging archetypes

Following analogous approaches in numerical taxonomy and ecology (Bezdek, 1974; Equiha, 1990; Salski, 2007), we used an established unsupervised algorithm (Dunn, 1973) to extract from our sample—based on the same features considered in Karagiannis et al. (2009)— a small number of fuzzy clusters, or *archetypes*. Archetypes—like usual clusters (called “crisp” in the context of fuzzy set theory)— are characterized by canonic properties, manifesting common phenotypic trends. In addition, as detailed in the section “Materials and Methods,” our analysis provided lists of *memberships* (Zadeh, 1965; Jang et al., 1997; Xu et al., 2008), quantifying the similarity of each neuron with different archetypes. Cells were considered as *archetypal*, if they had a large membership toward a unique archetype and as *atypical*, if they had comparable memberships toward more archetypes (Figure 1B).

Our clustering algorithm depends on few parameters, most notably on the fuzziness parameter μ (see Materials and Methods), the tuning of which affected the number of identified archetypes. For a sufficiently large fuzziness parameter, all cells were grouped indistinctly into a unique all-embracing fuzzy class. Decreasing the fuzziness, Glutamatergic neurons detached then first at μ = 1.81 and remained the only singularized archetype down to μ = 1.73 (Figures 2A,E). At this fuzziness level and down to μ = 1.63 a second class emerged (Figures 2B,E). Its GABAergic interneurons were PV-positive with electrophysiological properties reminiscent of FS interneurons and could be therefore identified as an archetype of FS-PV neurons (McCormick et al., 1985; Kawaguchi and Kubota, 1993).

At the fuzziness value of μ = 1.49 and down to μ = 1.37, a new GABAergic archetype differentiated (Figures 2C,E). It was mainly constituted by VIP-positive interneurons with an adapting firing pattern (Kawaguchi and Kubota, 1996; Cauli et al., 1997, 2000) and thus corresponded to the Adapting VIP archetype. Furthermore, within this same range of μ, the Glutamatergic type started differentiating into further subtypes.

In the range of 1.37 ≥ μ ≥ 1.21 two further archetypes of mostly SOM- or Neuropeptide Y (NPY)-positive interneurons progressively emerged (Figures 2D,E). The GABAergic archetypes found in that range (Figure 2E) reproduced to a good extent the GABAergic classes obtained with the two independent crisp clustering algorithms we previously used in Karagiannis et al. (2009), confirming their reliability. In the obtained soft hierarchy of fuzzy archetypes, a fuzzy class encompassing all neurons with a yet uncharacterized main type was found at any μ and was denoted as the class of “Unidentified Firing Objects” (UFOs).

Finally, classifications with a larger number of archetypes could not be robustly extracted from our dataset, since these partitions were valid only for very narrow ranges of μ (Figures 2E,F). In the rest of this study, we focus on the specific classification obtained for μ = 1.349, leading to four GABAergic archetypes, together with one (out of three merged) Glutamatergic archetype and a residual UFO class (Figures 2D,E).

Weighting features by their archetype memberships limits the confounding contribution of atypical cells, so our fuzzy method allowed for a better quantitative characterization of the typical features of these archetypes (summarized in Tables 1–4).

### Three glutamatergic subclasses

The whole population of Glutamatergic neurons was distinctly characterized by relatively deeply located somata, hyperpolarized resting membrane potentials, and slow time constants due to large membrane capacitances (Table 1). Their action potentials were of long duration with slowly developing afterhyperpolarization (AHP) of small amplitude (Table 3). When strongly depolarized these neurons fired action potentials with a marked amplitude accommodation, a pronounced frequency adaptation, and were unable to sustain high firing rates (Table 2). As previously described for glutamatergic neurons (Kubota et al., 1994; Andjelic et al., 2009), they expressed vGluT1 and only infrequently interneuronal markers, except for CB (Table 4). Although these neurons were the first to singularize, they subsequently split up into three subclasses at μ values required for the emergence of Adapting VIP, SOM, and NPY archetypes (Figures 2C–E). These classes of glutamatergic neurons only differed by the laminar location of their soma and by electrophysiological properties, as reported in Table 5.

**Table 5:** **Significant comparisons between glutamatergic subtypes**.

	**Glutamatergic class A**	**Glutamatergic class B**	**Glutamatergic class C**
	**(*n* = 25)**	**(*n* = 12)**	**(*n* = 9)**
(1) Digitized laminar location (*Layer*)	3.7 ± 0.6	**2.5 ± 0.8**	4.0 ± 0.2
	**Class B** < < Classes A, C		
(4) Time constant (τ_m_, ms)	33.4 ± 8.1	**42.6 ± 11.7**	34.2 ± 9.2
	Class A < **Class B**		
(6) Sag index (*Sag*, %)	21.5 ± 7.5	**11.3 ± 7.7**	25.8 ± 7.6
	**Class B** < < Classes A, C		
(9) Just-above threshold adaptation (Ad_thr_, Hz/s)	−18 ± 35	−4.7 ± 3.0	**−163 ± 76**
	**Class C** < < < Classes A, B		
(10) Minimal steady state frequency (*F*_min_, Hz)	14 ± 18	**7.9 ± 2.8**	**77 ± 15**
	Classes A, B < < < **Class C**		
(11) Amplitude accommodation (*A*_hump_, mV)	25.2 ± 6.2	**15.6 ± 6.9**	**33.2 ± 8.0**
	**Class B** < < Class A < **Class C**		
(13) Time constant of early adaptation (τ_sat_, ms)	23.6 ± 4.8	20.1 ± 3.5	**38 ± 18**
	Classes A, B < < < **Class C**		
(14) Late adaptation (Ad_sat_, Hz/s)	−9.7 ± 6.6	−14.5 ± 8.4	**−3.7 ± 3.8**
	Classes A,B < < **Class C**		
(15) Maximal steady state frequency (*F*_max_, Hz)	30.5 ± 8.6	32.6 ± 6.8	**23.2 ± 6.4**
	**Class C** < < Class B		
(17) Second spike amplitude (*A*_2_, mV)	90.4 ± 7.8	89.1 ± 9.3	**69.5 ± 5.2**
	**Class C** < < < Classes A, B		
(18) First spike duration (*D*_1_, ms)	1.40 ± 0.17	**1.60 ± 0.18**	1.41 ± 0.17
	Class A < < < **Class B**		
(20) Amplitude Reduction (Δ(Amp), %)	−3.7 ± 2.0	−2.9 ± 1.9	**−24.8 ± 3.9**
	Classes A,B < < < **Class C**		
(21) Duration Increase (Δ(Dur), %)	3.4 ± 2.8	8.3 ± 4.1	**31.3 ± 9.9**
	Class A < < Class B < < < **Class C**		
(22) First spike, first component AHP (AHP_f_, mV)	−5.5 ± 2.6	**−10.1 ± 4.5**	−5.4 ± 2.5
	**Class B** < Classes A, C		
(23) First spike, second component AHP (AHP_s_, mV)	**−13.6 ± 2.8**	−3.5 ± 6.0	0.0 ± 0.0
	**Class A** < < Classes B, C		
(24) Second spike, first component AHP (AHP_f,2_, mV)	−6.9 ± 2.6	−13.6 ± 4.2	−7.4 ± 2.8
	**Class B** < Classes A, C		
(25) Second spike, second component AHP (AHP_s,2_, mV)	−18.2 ± 2.2	**−0.7 ± 3.7**	**−23.0 ± 2.0**
	**Class C** < < < Class A < < < **Class B**		
(26) First spike, first AHP component latency (τ_AHPf_, ms)	5.3 ± 1.5	**42.6 ± 25.5**	4.5 ± 0.6
	Classes A,C <<< **Class B**		
(27) First spike, second AHP component latency (τ_AHPs_, ms)	**78.2 ± 23.7**	18.8 ± 33.7	0.0 ± 0.0
	Classes B,C <<< **Class A**		
(28) Second spike, first AHP component latency (τ_AHPf,2_, ms)	6.1 ± 1.6	**57.5 ± 15.1**	7.7 ± 1.7
	Class A < Class C <<< **Class B**		
(29) Second spike, second AHP component latency (τ_AHPs,2_, ms)	73.3 ± 17.0	**2.2 ± 12.5**	95.3 ± 23.1
	**Class B** <<< Class A < Class C		
(30) first spike ADP (ADP, mV)	**2.5 ± 1.8**	0.1 ± 0.3	0.0 ± 0.0
	Classes B,C <<< **Class A**		
(31) Second spike ADP (ADP_2_, mV)	0.4 ± 0.5	**0.01 ± 0.07**	0.3 ± 0.5
	**Class B** < Classes A, C		
(32) First spike ADP latency (τ_ADP_, ms)	**10.5 ± 4.3**	0.6 ± 2.5	0.0 ± 0.0
	Classes B,C <<< **Class A**		
(33) Second spike ADP latency (τ_ADP2_, ms)	4.6 ± 3.9	0.2 ± 1.4	5.1 ± 5.3
	**Class B** < Classes A, C		

*Values are weighted means ± SD; n, number of cells; < significantly smaller with P ≤ 0.05; << significantly smaller with P ≤ 0.01; <<< significantly smaller with P ≤ 0.001*.

Briefly, *class A glutamatergic neurons* whose somata were deeply located (Table 5) displayed a pronounced voltage sag at hyperpolarized potentials (Table 5). They distinctly differed from the other glutamatergic neurons by a first action potential exhibiting a very slowly developing and deep secondary AHP. Compared with other glutamatergic neurons the duration of their spikes was fairly constant (Table 5).

*Class B glutamatergic neurons* were the most superficial group of glutamatergic neurons and exhibited modest voltage sag when hyperpolarized (Table 5). The profile of their spike amplitude accommodation was of small amplitude for a glutamatergic neuron (Table 5). Their action potentials were of long duration and their first component AHP was slowly developing and of relatively large amplitude (Table 5).

Similarly to class A glutamatergic neurons, *class C glutamatergic neurons* had deeply located somata and a very pronounced voltage sag (Table 5). In contrast to the other glutamatergic neurons, none of them were able to fire below a minimal frequency and displayed strong adaptation when depolarized just above threshold (Table 5), two firing features reminiscent of a bursting phenotype (Hodgkin, 1948). Their first, but not their second action potential displayed a monophasic repolarization. The duration and the amplitude of their second action potential were respectively longer and smaller than those of their first spike. Also consistent with a bursting phenotype they displayed a very pronounced amplitude accommodation when strongly depolarized. Altogether these features indicate that class C glutamatergic neurons correspond to intrinsically bursting neurons (McCormick et al., 1985; Connors and Gutnick, 1990).

From the different properties available for all neurons in the dataset, it was not possible to associate other distinctive properties to these classes of glutamatergic neurons. They included molecular (Table 5) and basic morphological somatic features (data not shown). The absence of relevant markers for these neurons is attributable to the low occurrence of interneuron markers in glutamatergic neurons (Andjelic et al., 2009). In addition, the difficulty to correlate unambiguously the morphology of spiny granular and supragranular neurons with a firing pattern (McCormick et al., 1985; Connors and Gutnick, 1990; Staiger et al., 2004) further supports the idea that the three classes of glutamatergic neurons do not differ by their somatic features. Nevertheless, it is possible that these neurons exhibit distinctive patterns of dendritic and/or axonal arborizations, as it has been observed for bursting and non-bursting Layer V pyramidal cells (Chagnac-Amitai et al., 1990; Christophe et al., 2005). Since such properties are not available in our dataset, glutamatergic neurons will be considered as a single unified archetype in the rest of this study.

### Four GABAergic archetypes

Archetypal *FS-PV interneurons* had a very low input resistance, a very fast membrane time constant, and displayed the lowest electrical excitability (Tables 1 and 3, see also cell *a* in Figure 3 as an illustrative example). Their spikes were short with fast and deep monophasic AHP (Table 3) and they were able to sustain very high firing frequencies (Table 2). They expressed the GABA synthesizing enzymes GAD65 and/or 67, (GADs) and PV, frequently Calbindin (CB) and, to a lesser extent, NPY (Table 4).

Archetypal *Adapting VIP interneurons*, whose emergence followed that of FS-PV interneurons (Figure 2D), displayed a high resistance and a very small membrane capacitance (Table 1, see also cell *g* in Figure 4). Their spike waveform was complex with a fast AHP, followed by a marked afterdepolarization (ADP) and a secondary AHP (Table 3). At high stimulation, they showed a fast early adaptation followed by a marked late adaptation (Table 2). They expressed GADs and VIP and, relatively frequently, Calretinin (CR) (Table 4).

Archetypal *Adapting SOM interneurons* displayed a marked voltage sag in response to hyperpolarization (Table 1, see also cell *k* in Figures 3, 4). They were the most excitable neuronal archetype with a depolarized resting potential and a low rheobase (Table 2). Similarly to *Adapting VIP interneurons* their complex spike waveform was characterized by a prominent ADP (Table 3). At strong stimulation intensities, they very slowly adapted their firing frequency (Table 2). They expressed GADs and SOM and, often, CB and NPY (Table 4) (Kawaguchi and Kubota, 1996; Cauli et al., 2000; Karagiannis et al., 2009).

Archetypal *Adapting NPY interneurons* were weakly excitable, although less than the FS-PV archetype (Table 2, see also cell *o* in Figures 3, 4), and characterized by the virtual absence of adaptation near threshold (Table 2). They fired rather wide spikes with a very low minimal firing frequency (Table 3). Repolarization behavior consisted in a biphasic AHP with a slow first component and virtually no ADP (Table 3). They expressed GADs, often NPY and, relatively frequently, the neuronal isoform of nitric oxide synthase (NOS-1) (Table 4).

### Walks through diversity

Neurons can be represented as points in a high-dimensional space with coordinates given by their archetype memberships *m*_α_. For the purpose of visualization, positions of individual cells were plotted in different 3-dimensional projections of the full membership space (Figures 3–5). Such geometric views allowed following cell sequences with properties gradually varying between archetypes. Archetypal cells had, by definition, a single dominant membership and were thus located nearby an apex. Conversely, atypical cells were lying in the tetrahedral volume defined by three different apices.

FS-PV neurons were the first archetype of interneurons to emerge when decreasing the fuzziness parameter (Figure 2). We first describe a sequence linking these neurons with other archetypes. Since no marked shared membership between the FS-PV and Adapting VIP archetypes could be found (as visible from Figures 2 and 6A), we only considered linking sequences with the Adapting SOM and NPY archetypes (Figure 3A). A closed loop linking cells of the Adapting VIP, SOM, and NPY archetypes was examined in another sequence (Figure 4A).

The electrophysiological behaviors of interneurons sitting on these paths of membership space are depicted in Figures 3, 4B and their selected features are reported in tables of Figures 3, 4C. The background color of table entries encodes different ranges of feature values, which are typical for different archetypes (Tables 1–4). Accordingly columns in Figures 3, 4C corresponding to the features of cells *a* (*m*_FS−PV_ = 97%), *g* (*m*_AdVIP_ = 94%), *k* (*m*_AdSOM_ = 79%), and *o* (*m*_AdNPY_ = 83%), archetypal for FS-PV, Adapting VIP, SOM, and NPY archetypes respectively are dominated by a single color. Archetypal cells could also exhibit extreme distinctive values of certain hallmark features. For instance, cell *g* had a very small voltage sag and cell *o* a very low excitability. Conversely and indicative of transition phenotypes (Figures 3, 4C), the features of cells progressively diverging from one archetype and converging to another, gradually lose the color code of their starting archetype to adopt the color code of the target one.

### Atypical cells related with the FS-PV archetype

Figure 3 highlights transition cells on sequences linking the FS-PV to the Adapting SOM and Adapting NPY archetypes, of which neurons *a*, *k*, and *o* are representative examples.

Cell *c* is an example of a transition between FS-PV (*m*_FS−PV_ = 29%) and Adapting SOM (*m*_AdSOM_ = 21%) archetypes (Table 6). This neuron displayed a relatively small resistance, a high firing rate with narrow spikes and PV expression characteristic of FS-PV cells. Unusual for FS-PV cells, it exhibited an action potential waveform with a fast and deep AHP followed by a small ADP. Also reminiscent of its secondary Adapting SOM membership, it had a slow time constant and a prominent voltage sag in response to hyperpolarizing currents. Neuron *b* had also transition phenotypes between the FS-PV and the Adapting SOM archetypes.

**Table 6:** **Membership values of cells in the sequences of Figures 2, 3, and 4**.

**Cell**	**Glutama-tergic**	**FS-PV**	**Adapt. SOM**	**Adapt. VIP**	**Adapt. NPY**	**UFO**	**Comments**
a	0.00	0.97	0.00	0.00	0.01	0.01	Archetypal (FS-PV)
b	0.05	0.39	0.16	0.13	0.14	0.12	Atypical (FS-PV)
c	0.05	0.29	0.21	0.14	0.13	0.17	Edge cell (FS-PV—Adapting SOM)
d	0.05	0.40	0.15	0.07	0.21	0.12	Atypical cell (FS-PV)
e	0.08	0.22	0.12	0.07	0.38	0.13	Atypical cell (Adapting NPY)
f	0.10	0.20	0.31	0.05	0.20	0.13	Edge cell (Ad. SOM—FS-PV—Ad. NPY)
g	0.01	0.00	0.02	0.94	0.02	0.01	Archetypal (Adapting VIP)
h	0.04	0.02	0.23	0.49	0.09	0.13	Atypical (Adapting VIP)
i	0.06	0.02	0.40	0.26	0.11	0.15	Edge cell (Adapt. SOM—Adapt. VIP)
j	0.04	0.03	0.48	0.18	0.14	0.12	Atypical (Adapting SOM)
k	0.05	0.01	0.79	0.06	0.05	0.04	Archetypal (Adapting SOM)
l	0.01	0.03	0.07	0.49	0.24	0.16	Atypical (Adapting VIP)
m	0.06	0.05	0.14	0.34	0.22	0.19	Edge cell (Adapt. VIP—Adapt. NPY)
n	0.13	0.02	0.09	0.28	0.34	0.14	Edge cell (Adapt. NPY—Adapt. VIP)
o	0.01	0.01	0.04	0.01	0.83	0.10	Archetypal (Adapting NPY)
p	0.08	0.04	0.22	0.10	0.42	0.14	Atypical (Adapting NPY)
q	0.04	0.09	0.24	0.13	0.35	0.14	Edge cell (Adapt. NPY—Adapt. SOM)
r	0.10	0.13	0.33	0.09	0.22	0.13	Edge cell (Adapt. SOM—Adapt. NPY)
s	0.06	0.15	0.04	0.23	0.15	0.38	“UFO” main type (Burst. VIP)
t	0.20	0.05	0.15	0.07	0.16	0.37	“UFO” main type
u	0.04	0.09	0.06	0.06	0.34	0.41	“UFO” main type

*Memberships evaluated at μ = 1.349, rounded at two decimals (note that, due to rounding, membership sums can deviate slightly from unit)*.

Cell *e* illustrates a transition between Adapting NPY (*m*_AdNPY_ = 38%) and FS-PV (*m*_FS−PV_ = 22%) archetypes (Table 6). Its maximal firing frequency at high stimulation intensities was moderate and it expressed NPY, consistent with its main type. Reminiscent of its secondary type it exhibited a small resistance, a low electrical excitability, fired narrow spikes and expressed PV. Most other features assumed values in ranges compliant with both the Adapting NPY and the FS-PV archetypes except for its complex repolarization phase displaying an uncommon small ADP. Similarly, neuron *d* had transition phenotypes between the FS-PV and the Adapting NPY archetypes.

Finally, cell *f* illustrates a neuron sharing multiple memberships between Adapting SOM (*m*_AdSOM_ = 31%), FS-PV, and Adapting NPY (*m*_FS−PV_ = *m*_AdNPY_ = 20%) archetypes (Table 6). It had mostly intermediate properties but displayed a depolarized resting potential and voltage sag characteristic of Adapting SOM neurons, a short time constant, and deep monophasic repolarization reminiscent of its FS-PV membership. However, its AHP latency was rather long for a FS-PV neuron and was closer to that of Adapting NPY cells. Notably, it expressed the three distinctive molecular markers, PV, SOM, and NPY of its parent archetypes.

### Atypical cells related with the adapting VIP archetype

Figure 4 depicts another loop sequence linking the Adapting VIP, SOM, and NPY archetypes, of which neurons *g*, *k*, and *o* are representative examples.

Cell *i* is an illustrative example of a transition between Adapting VIP (*m*_AdVIP_ = 26%) and Adapting SOM archetypes (*m*_AdSOM_ = 40%, Table 6). Consistent with its Adapting SOM main type, it was fairly depolarized at rest, displayed a marked voltage sag, and slowly adapted at saturating frequencies (τ_sat_ = 34.0 ms, see Table 2). Indicative of its secondary membership, its membrane capacitance was rather small and it did not express SOM but VIP. Cells *h* and *j* had also mixed Adapting VIP and SOM traits.

Neuron *m* depicts a transition cell between Adapting VIP (*m*_AdVIP_ = 34%) and Adapting NPY (*m*_AdNPY_ = 22%) archetypes (Table 6). Reminiscent of its main type, it displayed a noticeable ADP, followed by a second component of AHP. It was also able to discharge at a large firing frequency (*F*_max_ = 120.9 Hz, cf. Table 2). Compatible with its secondary, but not with its main type, it exhibited a small resistance, a large rheobase and a long first spike latency (*t*_1st_ = 478.4 ms, see Table 2). Cells *l* and *n* had also transition phenotypes between the Adapting VIP and NPY archetypes.

### Atypical cells between the adapting SOM and NPY archetypes

In addition to the atypical cells with noticeable Adapting SOM or NPY memberships already described in the previous two sections and related as well with the FS-PV or Adapting VIP archetypes, Figure 4 shows other atypical cells lying between the Adapting SOM and NPY archetypes.

As an example, cell *q* had Adapting NPY main type (*m*_AdNPY_ = 35%) and a second important Adapting SOM membership (*m*_AdSOM_ = 24%, Table 6). Consistent with its main type it was weakly excitable and expressed NPY. It displayed a complex spike waveform with uncommon features such as a slow ADP, suggestive of an Adapting SOM phenotype, combined with a marked and fast second component of AHP, characteristic of Adapting NPY cells. In agreement with its secondary but, intriguingly, not with its main type (Tables 1–4), cell *q* had an intermediate minimal firing frequency and expressed CB. Cells *p* and *r* also exhibited mixed Adapting NPY and SOM traits.

### Other atypical cells

Finally, some highly atypical cells displayed heterogeneous phenotypes that could not be simply described as transitional between defined archetypes. All these cells had a large membership toward the residual UFO class. Neurons *s*, *t*, and *u* are illustrative example UFOs illustrated in Figure 5.

Despite being GABAergic, Cell *t* (*m*_UFO_ = 37%) displayed anomalous features, e.g., broad spikes and an inability to discharge at high firing rates, which are reminiscent of the Glutamatergic archetype (*m*_Glut_ = 20%, Table 6). It shared as well properties with the FS-PV (small spike amplitude), the Adapting SOM (depolarized resting membrane potential and intermediate first AHP amplitude), and the Adapting NPY archetypes (slow first AHP component) and it had a membrane resistance extreme even for the Adapting VIP archetype.

Few cells with UFO main type (*n* = 18 out of 33) displayed-marked bursting phenotype, defined by the inability to fire below a minimal frequency (Hodgkin, 1948) and characterized by strong near-threshold adaptation and spike amplitude reduction at the onset of firing. This hinted at the potential emergence of a further Bursting VIP archetype (Karagiannis et al., 2009) out of the heterogeneous and yet indistinct collection of cells constituting the UFO class. Cell *s* represents here a characteristic example of this tendency (Figure 5) and was indeed classified as belonging to the Bursting VIP type in the previous crisp analysis (Karagiannis et al., 2009).

### Graded and structured separations between archetypes

These partial lists of atypical cells suggest that, with the exception of Glutamatergic, FS-PV, and Adapting VIP archetypes (Figure 2), a neat separation between archetypes may be lacking. Figure 6A allows visualizing in a systematic way, through a series of 2-dimensional cross-sections, how neurons were scattered in the membership space. The two axes of every triangular panel in Figure 6A correspond to memberships toward a different pair of archetypes for each panel. The black dashed diagonal line corresponds to equal membership toward the two considered archetypes and it is surrounded by a shaded gray region corresponding to the halved mean absolute deviation. Cells falling within this region had therefore almost identical memberships to the two archetypes and were lying precisely at the geometric frontier between them. Consequently, we denoted them as *edge cells* (see Materials and Methods for definition).

There were no Glutamatergic cells at the edge with well-defined archetypes (i.e., any archetype apart from the UFO class), corresponding to a complete separation of the Glutamatergic archetype from all the other GABAergic archetypes. The separation between the FS-PV and the Adapting VIP archetypes, which were the first two clusters of GABAergic neurons emerging with decreasing value of μ (Figures 2B,C,E), was also very distinct and there was no edge cell between them.

Edge cells were found however at the fringe of other Adapting Archetypes. Considering Adapting SOM neurons, there were edge cells between the Adapting SOM and the FS-PV archetypes (*n* = 3) and between the Adapting SOM and the Adapting VIP archetypes (*n* = 5). Regarding Adapting NPY neurons, there were edge cells between the Adapting NPY and the FS-PV archetypes (*n* = 2) and between the Adapting NPY and the Adapting VIP archetypes (*n* = 4). There was also a conspicuous overlap between the two Adapting SOM and NPY archetypes themselves (*n* = 8 edge cells). Finally, every archetype had cells at the edge with the UFO class, but these cells could not easily be described as transitional, since there is no clear phenotype associated to the UFO class.

To achieve an accurate and synthetic description of cell distribution in a high-dimensional space, we quantified for each cell its distance from the boundary between its main and secondary archetypes by computing its *typicality coefficient d*. As defined in the *Materials and Methods*, cells with typicalities larger or smaller than specific thresholds, related to the halved mean absolute deviation of the typicality distribution, were considered respectively as archetypal or atypical. The chosen thresholds captured well the bimodal structure of the sample distribution of *d* at μ = 1.349, in which peaks associated to strictly archetypal and atypical cells were clearly visible, as shown in Figure 6B.

According to the used criteria, 40% of the cells (*n* = 80 of 200) resulted as archetypal and 42% (*n* = 83 out of 200) as atypical. However, the distribution of archetypal and atypical cells was not homogeneous across different archetypes. The Glutamatergic, the FS-PV and, to a lesser extent, the Adapting VIP archetypes, were well-detached from the other archetypes, as indicated by their large average typicality coefficients and fractions of strictly archetypal cells (Figure 6). Other GABAergic archetypes displayed larger strictly atypical fractions. UFOs were an exceptional case, since none of them were archetypal (see Figure 6B and Table 6).

We note that the actual distribution of typicality coefficients depended on the chosen value of the fuzziness parameter. However, since the relative degrees of typicality of different cells were very well-preserved over a wide range of parameters, the fractions of cells labeled as atypical or archetypal were robustly conserved (not shown).

### The relevance of different features for classification

The archetypes and the typicality spectrum observed in our sample reflected the existence of structured correlations between the values of different features in different cells. A first natural question is therefore establishing which of the measured features are the most decisive for the determination of the extracted fuzzy partition. A second question is whether it would be possible to use fewer features and, yet, obtain an equivalent classification.

Generalizing an approach we previously introduced (Karagiannis et al., 2009), we assessed the relevance for classification of different properties or groups of properties by means of partial randomization analyses. The values of a subset of properties were “scrambled” by permuting them randomly between different cells. Such a manipulation preserved by construction the sample average and standard deviation of the scrambled features, but destroyed their correlation with other features. Scrambling properties of higher significance to the observed fuzzy partition will lead to a lower classification quality. We quantified such variations in quality of fuzzy clustering by comparing the *cost function* of the extracted fuzzy partitions (see Materials and Methods) before and after randomization.

The increase in classification cost determined by randomization of individual features varied in the 2–4% range. Properties are ranked in Table 7 in descending order of cost increase after individual randomization. GAD expression (feature 35, Table 4), discriminating excitatory and inhibitory neurons, was the property associated to the largest cost increase. It was, followed by the maximum steady state frequency *F*_max_ (feature 15, Table 3), distinctly high for FS-PV neurons and very low for Glutamatergic neurons (Table 2). Note that the first two archetypes to emerge when decreasing the fuzziness μ were the Glutamatergic and the FS-PV archetypes.

**Table 7:** **Relevance of properties for the classification of our sample**.

**Rank**	**Feature**	**Δ*J* (%)**
#1	(35) GAD	4.80 ± 0.60
#2	(15) Maximal steady state frequency	4.46 ± 0.70
#3	(22) First spike, first component AHP	4.45 ± 0.75
#4	(18) First spike duration	4.41 ± 0.72
#5	(19) Second spike duration	4.41 ± 0.65
#6	(11) Amplitude accommodation	4.21 ± 0.68
#7	(24) Second spike, first component AHP	4.01 ± 0.76
#8	(27) First spike, second AHP component latency	3.96 ± 0.70
#9	(32) First spike ADP latency	3.69 ± 0.70
#10	(31) Second spike ADP	3.65 ± 0.81
#11	(34) VGluT1	3.63 ± 0.77
#12	(29) Second spike, second AHP component latency	3.59 ± 0.74
#13	(30) first spike ADP	3.55 ± 0.80
#14	(4) Time constant	3.54 ± 0.78
#15	(14) Late adaptation	3.46 ± 0.76
#16	(41) VIP	3.44 ± 0.76
#17	(38) PV	3.37 ± 0.82
#18	(33) Second spike ADP latency	3.35 ± 0.86
#19	(21) Duration Increase	3.35 ± 0.67
#20	(23) First spike, second component AHP	3.33 ± 0.72
#21	(12) Amplitude of early adaptation	3.33 ± 0.84
#22	(7) Rheobase	3.31 ± 0.75
#23	(16) First spike amplitude	3.27 ± 0.80
#24	(25) Second spike, second component AHP	3.20 ± 0.82
#25	(5) Membrane capacitance	3.19 ± 0.78
#26	(17) Second spike amplitude	3.18 ± 0.71
#27	(20) Amplitude reduction	3.16 ± 0.66
#28	(3) Input Resistance	3.10 ± 0.85
#29	(26) First spike, first component AHP latency	3.07 ± 0.65
#30	(40) NPY	3.06 ± 0.79
#31	(9) Just-above threshold adaptation	3.06 ± 0.63
#32	(6) Sag index	3.03 ± 0.76
#33	(8) First spike latency	2.94 ± 0.76
#34	(28) Second spike, first component AHP latency	2.89 ± 0.98
#35	(42) SOM	2.88 ± 0.76
#36	(2) Resting Potential	2.85 ± 0.80
#37	(1) Digitized laminar location	2.85 ± 0.70
#38	(10) Minimal steady state frequency	2.76 ± 0.69
#39	(43) CCK	2.73 ± 0.83
#40	(13) Time constant of early adaptation	2.69 ±0.75
#41	(39) CR	2.69 ± 0.95
#42	(37) CB	2.58 ± 0.78
#43	(36) NOS1	2.45 ± 0.91

*Increase of the residual cost of the fuzzy partition estimated via single-property randomization. Mean values and sample standard deviations are taken over 1000 independent randomizations*.

**Figure 7:**
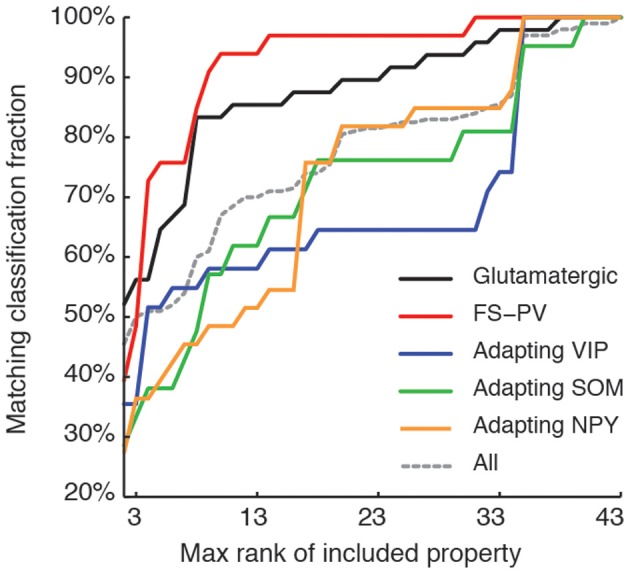
**Relevance of features for classification.** Matching between classifications based on a reduced number of top-ranked properties with the reference classification based on all the 43 features (see Table 7 for relevance ranking of the different properties). Classification matching is analyzed separately for every well-defined archetype and is measured by the fraction of cells with a given main type, matching in both the reference, and a reduced classification. Matching classification fraction for all archetypes confounded is also shown for comparison. Note the order of correctly classified archetypes corresponding to the historically characterized neuronal types.

We next sought to determine whether it was possible to reproduce the reference classification with a parsimonious list of features (Figure 7). We, thus, generated different classifications, starting with the most relevant property (i.e., the expression of GAD according to the ranking in Table 7) and then gradually adding new properties in decreasing order of relevance (see Materials and Methods). We found that while certain archetypes could be well-discriminated by a small number of top-ranked features, the correct discrimination of other archetypes required the consideration of a much larger number of properties. About half of the neurons with Glutamatergic or FS-PV main type could be identified by considering only the top three-ranked properties, i.e., GAD expression, maximum steady state frequency *F*_max_ and first AHP component of the first spike AHP_f_ (feature 22, Table 3). Considering also the duration *D*_1_ of the first and *D*_2_ of the second spikes (features 18–19, Table 3), allowed the correct classification of over half of the neurons with Adapting VIP main type and raised the fraction of correctly identified FS-PV neurons to over 70%. At least nine features were required for the identification of more than half of the cells with Adapting SOM main type, and at least 13 features for the identification of more than half of the cells with Adapting NPY main type. Correct identification of over 90% of all cells in the dataset was only possible by considering at least 35 features, the 30th and the 35th of which being respectively the molecular markers NPY and SOM.

Interestingly, this observed order of correctly classified archetypes is similar to the order of emergence of archetypes obtained by decreasing the fuzziness parameter (Figure 2) as well as with their historical identification (Mountcastle et al., 1969; McCormick et al., 1985; Kawaguchi and Kubota, 1993, 1996; Karagiannis et al., 2009).

It should be however noted that the results of these property relevance analyses are strongly dependent on the fine structure of our sample. Furthermore, the effects of randomizing more than one property simultaneously cannot be predicted trivially by the knowledge of the effects of randomization of individual properties. For instance, individual randomization of SOM and NPY expression have a small overall impact on classification, but their joint randomization can strongly affect the correct detection of Adapting SOM and NPY archetypes (not shown). More systematic analyses over multiple independent samples would thus be required to make general statements on the impact on classification of different properties or property groups.

## Discussion

### A structured continuum

Neuronal features varied in a remarkably graded way. However, this landscape of diversity was far from being a continuum without structure, since definite trends and trend changes were evident along archetype transitions (Figures 3–4).

Our approach allowed describing this structured continuum of phenotypes, by mediating between contrasting philosophies of neuronal classification (Tyner, 1975; Parra et al., 1998). On the one hand, we avoided the provocative view of the existence of a separate class for each cell (Parra et al., 1998). On the other hand, we could preserve information about the observed phenotypic diversity, which subdivisions into sharply distinct classes (Markram et al., 2004; Burkhalter, 2008) would have curtailed.

Unlike possibly more powerful but unavoidably more abstract dimensionality reduction techniques (Bishop, 2006; Hinton and Salakhutdinov, 2006), fuzzy memberships had a straight biological relevance, representing directly and compactly the similarity between single cells and canonic template archetypes. Because of an intuitive spatial representation of sample diversity, the analysis of memberships allowed thus the systematic detection of atypical and archetypal cells within a dataset already too large to be parsed without neuroinformatic assistance.

### The “right” number of archetypes

The four reference GABAergic archetypes and the typicality spectrum which we found at μ = 1.349 were robust in a wide parameter range. They were also optimal, in the sense that any attempt to reliably extract more archetypes from our sample was unsuccessful. Indeed, for μ < 1.204, even tiny changes in μ shattered entirely the extracted fuzzy partition (Figure 2E). Introducing a metaphor, one might say that adopting a too small μ would be like looking at a poor quality printed image through a macro lens. This would reveal most likely the low resolution of the halftone screen, rather than additional relevant details. Conversely, using a too large μ would be like taking an out-of-focus snapshot (Figure 2F). Therefore, our present list of archetypes should be considered, citing again Tyner (1975, p. 91), as “a temporary convenience, a rather crude data storage system,” reflecting only our present state of knowledge (and ignorance).

The failure to extract more archetypes was likely due to the lack of supplemental properties allowing subdivision of neuronal populations (Tyner, 1975). Such features might correspond to morphology and connectivity patterns (Markram et al., 2004; Christophe et al., 2005; Krimer et al., 2005; Perin et al., 2011), synaptic activity (Dumitriu et al., 2007), temporal dynamics of activity (Klausberger and Somogyi, 2008), and/or mechanisms of synaptic plasticity (Szabo et al., 2012). The analysis summarized by Figure 7 revealed that a smaller number of features was required to correctly discriminate the archetypes that emerged earlier at a high value of μ. The consideration of additional features in our classification would therefore most likely allow the discrimination of additional neuronal types, not yet emerged out of the residual UFO class. The simple results summarized by Figure 7 and Table 7 constitute only the first steps of a systematic study of the impact of different properties on neuronal classification, which goes beyond the limits of the present study.

Finally, since specific selection criteria (i.e., superficial layers and prominent radial processes) were used to increase the probability of collecting NPY neurons (Karagiannis et al., 2009), our results are not necessarily representative of the entire population of cortical interneurons. Nevertheless, in a complementary study aimed at characterizing deep layers neurons not necessarily exhibiting radial processes (Perrenoud et al., 2013), very similar classes of interneurons were obtained. Therefore, we believe that our approach provides at least a comprehensive view of interneuronal types, even if it does not claim to give an absolute and definitive classification of them.

### From development to graded diversity

Neuronal phenotypes are largely determined by developmental processes, which depend not only on the embryonic origin and date of birth (Butt et al., 2005; Miyoshi et al., 2007) but also on the signaling environment (Batista-Brito and Fishell, 2009) or the ongoing network activity (Cossart, 2011).

Glutamatergic neurons have embryonic origins, dates of birth, and migration patterns very different from that of interneurons (Wonders and Anderson, 2006). They were the first archetype to singularize and had no edge cell with the four well-defined interneuron archetypes (Figures 2, 6). Similarly, the FS-PV and the Adapting VIP archetypes exhibiting also clearly distinct embryonic origins (Butt et al., 2005; Miyoshi et al., 2007; Lee et al., 2010; Vucurovic et al., 2010) were the two first interneuron archetypes to emerge and no edge cell could be found between them. These observations suggest that neuronal archetypes that are primarily specified by clearly distinct spatial and temporal origins generally display very different phenotypic traits (Wonders and Anderson, 2006). Such multiple distinctive features result in an early singularization and therefore in a limited number of edge cells.

Neurons initially sharing similar origins progressively specify during development to form distinct neuronal classes. FS-PV and Adapting SOM neurons, both derived from the medial ganglionic eminence (Wonders and Anderson, 2006; Miyoshi et al., 2007), provide an example of such a developmental divergence. The observation of edge cells between these archetypes could speculatively reflect a vestige of their shared spatial embryonic origin. Alternatively, since neurons were collected from juvenile rats (P19) the existence of edge cells between FS-PV and Adapting SOM neurons (e.g., cell b) which exhibited a relatively high input resistance could also correspond to maturating FS-PV neurons. Indeed, these neurons progressively reduce their input resistance by acquiring two-pore K^+^ leak conductances (Okaty et al., 2009).

Conversely, neurons of distinct embryonic origins can also converge to a similar functional class. Adapting NPY neurons provide such an example of developmental convergence. Although they display common electrophysiological, molecular, and morphological features (Povysheva et al., 2008; Gelman et al., 2009; Karagiannis et al., 2009; Zaitsev et al., 2009; Lee et al., 2010) they remarkably originate either from the medial or caudal ganglionic eminences, or the preoptic area (Gelman et al., 2009; Tricoire et al., 2010; Vucurovic et al., 2010).

### From local cues to graded diversity

The overall converging phenotypic homogeneity of Adapting NPY neurons is however mitigated by the differential expression of NOS-1 (Cauli et al., 2004; Karagiannis et al., 2009; Kubota et al., 2011; Perrenoud et al., 2012a), which in the hippocampus (Tricoire et al., 2010; Jaglin et al., 2012), but not in the neocortex (Magno et al., 2012), is dependent on the embryonic origins.

This hints at other potential determinants of graded diversity. The density of cortical NOS-1 interneurons is enriched in the vicinity of blood vessels (Cauli et al., 2004; Tricoire et al., 2010; Perrenoud et al., 2013; Rockland and Nayyar, 2012). Given the shared local cues governing neuritic branching and vascular sprouting (Stubbs et al., 2009; Adams and Eichmann, 2010), it is likely that the local vascular environment might also orientate phenotypic traits.

More generally, an appealing hypothesis deserving future investigation is that certain atypical traits could emerge as a phenotypic adaptation to context-dependent signaling. The acquisition of secondary functional specializations would contribute to blur inter-archetype boundaries.

### A reservoir of phenotypes for functional degeneracy

An important motivation underlying many studies of neuronal diversity is to establish a correspondence between neuronal types and their respective functions. Under this perspective, the diversity of neuronal types would correspond tightly to the multiplicity of possible functional specializations (Burkhalter, 2008). Such a view is however oversimplified under many aspects.

First, relatively homogeneous groups of neurons can exert multiple functions. For instance, the overall population of Adapting NPY/neurogliaform neurons (Karagiannis et al., 2009; Zaitsev et al., 2009) is responsible for slow GABAergic inhibition (Tamás et al., 2003; Szabadics et al., 2007; Oláh et al., 2009). Since NPY and NO are respectively potent vasoconstrictor and vasodilator of cortical blood vessels (Cauli et al., 2004; Cauli and Hamel, 2010), the Adapting NPY archetype might also be specialized in a complex control of blood perfusion (Estrada and DeFelipe, 1998).

Second, different neuronal populations can contribute and/or cooperate to a same function. The neurovascular coupling is indeed mediated by multiple vasoactive messengers produced by different cell types (Attwell et al., 2010; Cauli and Hamel, 2010). Several neuronal types, including NOS-1 and VIP-expressing interneurons (Cauli et al., 2004; Perrenoud et al., 2013) as well as COX-2 expressing pyramidal cells (Niwa et al., 2000; Wang et al., 2005; Lecrux et al., 2011) are the primary sources of these vasodilatory compounds. Such a partial functional overlap between different neuronal types confers robustness to the neurovascular response (Leithner et al., 2010), essential for normal brain functions (Iadecola, 2004).

Therefore, the will to maintain a one-to-one matching between neuronal types and functions would ineluctably lead to the definition of a combinatorial number of neuronal types (Parra et al., 1998).

The multiplicity of cell types devoted to the neurovascular response constitutes an example of *functional degeneracy* (Edelman, 1987; Tononi et al., 1999), defined as the ability of heterogeneous elements to perform the same function. Beyond *redundancy*, occurring when a given function is achieved by replicating homogeneous elements, *degeneracy* confers higher robustness through adaptability. Indeed, heterogeneous elements can react differently in different contexts providing a considerable margin of safety over a wide spectrum of conditions.

### A reservoir of phenotypes for functional compensation

Another interesting question raised by our systematic exploration is how specific functions could be reliably achieved by networks whose cellular components are heterogeneous in a structured and graded manner.

A same circuit function can arise from very diverse combinations of neuronal properties and interaction mechanisms (Prinz et al., 2004; Goaillard et al., 2009). Indeed, heterogeneity of cellular properties does not necessarily translate to different performance at the network level for at least two reasons: First, many of these properties might be irrelevant for the function of the network, and, in absence of specific constraints, they would freely assume values over broad ranges. Second, even variations of certain critical properties in a cell might be compensated by covariations in other cells in the network. These two aspects might contribute to the intra-archetype diversity emphasized by our analysis. In addition, compensation between heterogeneous elements might provide a more flexible and robust solution than optimization of individual elements to achieve network functions (Marder and Goaillard, 2006). In this context the existence of a structured continuum of diversity might be viewed as a prerequisite for the emergence of reliable operation at the system level.

### Conflict of interest statement

The authors declare that the research was conducted in the absence of any commercial or financial relationships that could be construed as a potential conflict of interest.
